# Lipoprotein-Associated Phospholipase A2 Is Associated with Atherosclerotic Stroke Risk: The Northern Manhattan Study

**DOI:** 10.1371/journal.pone.0083393

**Published:** 2014-01-09

**Authors:** Mira Katan, Yeseon P. Moon, Myunghee C. Paik, Robert L. Wolfert, Ralph L. Sacco, Mitchell S. V. Elkind

**Affiliations:** 1 Department of Neurology, Columbia University College of Physicians and Surgeons, New York, New York, United States of America; 2 Department of Neurology, University Hospital of Zurich, Switzerland; 3 Department of Biostatistics, Mailman School of Public Health, Columbia University New York, New York, United States of America; 4 diaDexus, Inc., San Francisco, California, United States of America; 5 Department of Neurology, Miller School of Medicine, University of Miami, Miami, Florida, United States of America; 6 Departments of Epidemiology and Human Genetics, Miller School of Medicine, University of Miami, Miami, Florida, United States of America; 7 Department of Epidemiology, Mailman School of Public Health, Columbia University, New York, New York, United States of America; Innsbruck Medical University, Austria

## Abstract

**Background:**

Lipoprotein-associated phospholipase A2 (LpPLA2) levels are associated with stroke, though whether this extends to all populations and stroke subtypes is unknown.

**Methods:**

Serum samples from stroke-free community participants in the Northern Manhattan Study were assayed for LpPLA2 mass and activity. Participants were followed annually for stroke. Cox-proportional-hazard models were fitted to estimate hazard-ratios and 95% confidence intervals (HR, 95% CI) for the association of LpPLA2 levels with ischemic stroke (IS), after adjusting for demographic and medical risk factors.

**Results:**

Serum samples were available in 1946 participants, of whom 151 (7.8%) experienced a first IS during median follow-up 11 years. Mean age was 69 (SD 10), 35.6% were men, 20% non-Hispanic Whites, 22% non-Hispanic Blacks, and 55% Hispanics. LpPLA2 mass and activity levels were not associated with overall IS risk.

LpPLA2 mass but not activity levels were associated with strokes due to large artery atherosclerosis (LAA; adjusted HR per SD 1.55, 95% CI 1.17–2.04). There was a dose-response relationship with LAA (compared to first quartile, 2nd quartile HR = 1.43, 95% CI 0.23–8.64; 3rd quartile HR = 4.47, 95% CI 0.93–21.54; 4th quartile HR = 5.07, 95% CI 1.07–24.06). The associations between LpPLA2-mass and LAA-stroke risk differed by race-ethnicity (p = 0.01); LpPLA2-mass was associated with increased risk of LAA among non-Hispanic Whites (adjusted HR per SD 1.44, 95% CI 0.98–2.11), but not other race-ethnic groups.

**Conclusion:**

LpPLA2-mass levels were associated with risk of atherosclerotic stroke among non-Hispanic White participants, but not in other race-ethnic groups in the cohort. Further study is needed to confirm these race-ethnic differences and the reasons for them.

## Introduction

Stroke is the fourth leading cause of death in the United States and the leading cause of functional impairment [1]. Because stroke is largely preventable, tailoring treatment based on estimated risk may target therapies to high-risk patients and reduce burden of disease. For this purpose, the measurement of soluble biomarkers, which indicate underlying subclinical pathological processes, could be an important adjunct to traditional risk assessment. A highly promising candidate biomarker for risk stratification is lipoprotein-associated phospholipase A2 (LpPLA2).

Experimental data indicates that LpPLA2 has important pro-inflammatory effects specifically affecting vessel walls [2]. Clinical data using intravascular ultrasound suggests that elevated levels and increased activity of LpPLA2 are related to the progression of atherosclerotic disease [3]. Levels of LpPLA2 mass and activity were strongly associated with increased risk of cardiovascular events (either MI or composite endpoints) in several large cohort studies and meta-analyses [4]. Darapladib, a potential therapeutic agent specifically targeting LpPLA2 activity, is currently under investigation as a treatment administered after acute coronary syndromes and for stable coronary disease [5], [6].

Compared to the plethora of studies concerning LpPLA2 and cardiovascular events, only a few population-based studies have specifically addressed the association of LpPLA2 mass and activity and ischemic stroke as a primary outcome. The available data suggests that among those without a history of stroke, the risk of stroke is approximately doubled among those with the highest levels of LpPLA2, compared to those with the lowest levels [7]. The risk of recurrent stroke is similarly elevated among those with a first stroke [8], [9].

We hypothesized that LpPLA2 levels would be associated with stroke risk in the stroke-free population of Northern Manhattan, and we further sought to determine whether associations differed by race-ethnicity and stroke subtype.

## Methods

### Ethic statement

This study received approval from the institutional review boards at Columbia University Medical Center and the University of Miami, and all participants directly or through a surrogate when appropriate, gave their written informed consent for the study.

### Participants

The Northern Manhattan Stroke (NOMAS) study is a prospective, population-based cohort study consisting of 3298 stroke-free multiethnic participants enrolled between 1993–2001. The primary goals of the study were to describe the prevalence of vascular risk factors and incidence of vascular outcomes in a community-based sample of a racially and ethnically diverse population, as previously described [10]. Briefly, participants were ≥40 years at enrollment and resided in northern Manhattan, New York. The race/ethnic distribution of this cohort consists of 63% Hispanics, 20% non-Hispanic Black, and 15% non-Hispanic White residents.

Participants underwent a thorough baseline examination including comprehensive medical history, physical examination, review of medical records, and fasting blood samples, as previously described [10]. Trained bilingual research assistants performed interviews; study physicians conducted physical and neurological examinations. To assess medical history, standardized questions were adapted from the Behavioral Risk Factor Surveillance System by the Centers for Disease Control and Prevention [11]. Race-ethnicity was self-identified by participants using standard census questions.

### Biomarker measurement

Blood samples collected at enrollment were centrifuged and frozen at −80°C in 1 ml aliquots until the time of analysis. For this analysis, samples were available in 1946 participants. The available samples were shipped on dry ice. On arrival they were thawed to 4°C prior to measurements and analyzed within one hour thereafter, thus reducing pre-analytic problems. Serum samples were assayed for levels of LpPLA2 mass, using a manual enzyme-linked immunoassay (PLAC), and LpPLA2 activity, using a colorimetric activity method (CAM), at a central laboratory at diaDexus, Inc., San Francisco, CA, USA. Quality control was maintained using standardized procedures including running samples in duplicate. All testing was performed in a batch analysis blinded to all clinical data including outcome.

### Prospective Outcome Assessment

Annual telephone follow-up assessed vital status as well as interval hospitalization or illness and specifically symptoms indicative of ischemic stroke; the average annual contact rate was 99% [12]. A positive screen for suspected ischemic stroke was followed up by in-person assessment to determine whether an outcome had occurred. We prospectively screened all admissions and discharges to detect hospitalizations and outcomes that may not have been captured by telephone interview. Stroke was defined as the first symptomatic occurrence of fatal and non-fatal ischemic stroke according to the World Health Organization criteria [13]. Ischemic stroke subtype was classified by a consensus of two study neurologists, with a third neurologist adjudicating if needed [14], based on the TOAST (Trial of Org 10172 in Acute Stroke Treatment) criteria [15].

### Statistical Analyses

Descriptive statistics were calculated, including means for continuous variables and proportions for dichotomous variables. The primary outcome for this analysis was ischemic stroke and the secondary outcome was large artery atherosclerotic stroke (LAA). The main predictors were LpPLA2 mass and activity considered as continuous predictors and also by quartiles in order to facilitate clinical interpretation. We fitted Cox proportional hazard models to calculate hazard ratios and 95% confidence intervals (HR, 95% CI), unadjusted and adjusted for demographic and vascular risk factors, including lipid levels. Adjusted covariates were predictors of ischemic stroke in prior analyses in NOMAS and traditionally accepted risk factors for stroke. We tested for interactions between LpPLA2 and sociodemographic and vascular risk factors. The interaction models were also fully adjusted using the same variables as in the Cox proportional hazard models. The final models were tested to ensure no violation of the proportionality assumption for the Cox model. We ran sensitivity analyses using the inverse probability weighted (IPW) method to correct for potential selection bias that may have been introduced when the LpPLA2 cohort were selected from the full NOMAS cohort. All testing was two-tailed and p<0.05 was considered to indicate statistical significance. All calculations were performed using SAS v9.1.3 (SAS Institute, Cary, NC).

## Results

### Study population characteristics

Among 3298 in the stroke free cohort, LpPLA2 levels were available for 1946 participants. The current study population consisted of 35.6% men; mean age was 69 (SD± 10) years, 20% were non-Hispanic Whites, 22% non-Hispanic Blacks, and 55% Hispanics. Mean LpPLA2 mass levels were 308.7 ng/mL (SD 88.2), and mean LpPLA2 activity levels 116.9 nmol/min/mL (SD 29.6). Characteristics among these participants were similar to those not in this analysis; this study cohort was slightly younger (mean age 69 versus 70), and participants were more likely to be female (64% versus 61%), Hispanic (55% versus 48%), or suffer from diabetes mellitus (24% versus 18%), and they were more likely to be physically inactive (55% versus 62%), and less likely to complete high school (44% versus 49%). We present all baseline characteristics according to race ethnicity (table 1).

**Table 1:** Baseline characteristics according to race ethnicity.

Sociodemographic factors	Whole cohort	Hispanic	Non-Hispanic White	Non-Hispanic Black	P-value§
N	1946	1059	381	425	
Age, mean ± SD (years)	69±10	66±9	73±10	72±11	<0.0001
Female sex, n (%)	1253 (64%)	682 (64%)	237 (61%)	300 (70%)	0.02
Education (High School graduate), n (%)	856 (44%)	222 (21%)	328 (85%)	266 (62%)	<0.0001
**Vascular risk factors**					
Hypertension*, n (%)	1447 (74%)	813 (76%)	253 (65%)	350 (82%)	<0.0001
Systolic blood pressure, mean ± SD (mmHG)	144±20	143±21	140±20	148±21	<0.0001
Coronary heart disease, n (%)	429 (22%)	218 (20%)	101 (26%)	97 (23%)	0.06
Diabetes mellitus, n (%)	467 (24%)	282 (26%)	56 (15%)	121 (28%)	<0.0001
Waist circumference, mean ± SD	36.8±4.9	37±5	36±5	37±5	0.006
Any physical activity, n (%)	1067 (55%)	521 (49%)	250 (64%)	261 (61%)	<0.0001
Past smoker, n (%)	674 (35%)	344 (32%)	158 (41%)	150 (35%)	<0.0001
Current smoker, n (%)	347 (18%)	179 (17%)	56 (14%)	103 (24%)	<0.0001
Moderate alcohol consumption**, n (%)	647 (33%)	322 (30%)	165 (43%)	139 (32%)	<0.0001
**Laboratory measurements**					
LpPLA2, mean ± SD (mass, ng/ml)	308.7±88.2	300.2±84.9	349.6±82.4	294.9±90.8	<0.0001
LpPLA2, mean ± SD (activity, nmol/min/mL)	116.9+29.6	114±27.7	131.4±31.0	109.1±28.1	<0.0001
HDL, mean ± SD (mg/dL)	46.5±14.2	43.5±12.9	48.6±14.3	51.1±15.2	<0.0001
LDL, mean ± SD (mg/dL)	129.2±35.5	129. 5±34.8	132.7±34.2	125.5±37.6	0.02

SD: standard deviation; IQR: interquartile range.

Hypertension defined as: history, taking medications, or systolic blood pressure ≥140 mmHg, or diastolic blood pressure ≥90 mmHg;

Moderate alcohol intake: 1 alcoholic drink per week to 2 drinks per day.

Kruskal Wallis test for continuous variables and Chi-Squared test for categorical variables.

### Association of LpPLA2 levels with incident ischemic stroke

Among the 1946 participants, 151 (7.8%) developed an ischemic stroke during a median follow-up of 11 years. Stroke etiologies according to the TOAST criteria were distributed as follows: 26 (17%) LAA, 35 (23%) small vessel, 54 (36%) cardioembolic, and 36 (24%) unknown etiology.

In the adjusted analysis, LpPLA2 mass (adjusted HR per SD 1.02, 95% CI 0.86–1.19) and activity (0.88, 95% CI 0.71–1.08) were not associated with ischemic stroke (see table 2). There was also no association when analyzing LpPLA2 mass and activity by quartiles.

**Table 2:** Association of LpPLA2 mass and activity with incident ischemic stroke.

Biomarker levels	Hazard Ratio	95%CI
**LpPLA2-mass (per SD):**		
unadjusted	1.03	0.89–1.21
adjusted for demographics*	0.99	0.84–1.16
fully adjusted**	1.02	0.86–1.19
**LpPLA2-activity (per SD):**		
unadjusted	0.92	0.78–1.01
adjusted for demographics*	0.83	0.69–0.99
fully adjusted**	0.88	0.71–1.08

age, gender, race-ethnicity, education.

adjusted for age, sex, race-ethnicity, education, waist circumference, physical activity, moderate alcohol consumption, smoking status, diabetes mellitus, systolic blood pressure, coronary artery disease, Low density lipoprotein, High density lipoprotein.

### Association of LpPLA2 and large artery atherosclerotic strokes (LAA)

LpPLA2 mass but not activity levels were associated with LAA (adjusted HR per SD 1.55, 95% CI 1.17–2.04). When analyzed by quartiles, there was a dose-response relationship: compared to the lowest quartile of LpPLA2 mass, there was an increased risk for those in the second (adjusted HR = 1.43, 95% CI 0.23–8.64), third (adjusted HR = 4.47, 95% CI 0.93–21.54), and fourth quartiles (adjusted HR = 5.07, 95% CI 1.07–24.06; table 3). LpPLA2 mass was not associated with cardioembolic stroke (adjusted HR per SD 0.91, 95% CI 0.69–1.20), or lacunar stroke (adjusted HR per SD 0.69, 95% CI 0.46–1.05).

**Table 3:** Association of LpPLA2 with large-artery atherosclerotic (LAA) stroke.

Biomarker levels	UnadjustedHR (95% CI)	Model 1*,HR (95% CI)	Model 2**,HR (95% CI)
LpPLA2-mass (per SD)	1.57 (1.26–1.96)	1.49 (1.18–1.88)	1.55 (1.17–2.04)
LpPLA2-activity (per SD)	1.34 (0.92–1.94)	1.15 (0.76–1.73)	1.17 (0.71–1.92)
LpPLA2-mass (the number of LAA, levels)			
Q1 (n = 2, 28.1–245.6)	Ref.	Ref.	Ref.
Q2 (n = 3, 245.7–307.2)	1.53 (0.26–9.15)	1.42 (0.24–8.52)	1.43 (0.23–8.46)
Q3 (n = 9, 307.2–365.5)	4.63 (1.00–21.44)	4.09 (0.88–19.12)	4.47 (0.93–21.54)
Q4 (n = 12, 365.5–972.6)	6.19 (1.39–27.64)	4.88 (1.06–22.45)	5.07 (1.07–24.06)
LpPLA2-activity (the number of LAA, levels)			
Q1 (n = 5, (12.1–96.5)	Ref.	Ref.	Ref.
Q2 (n = 4, 96.6–115.5)	0.69 (0.19–2.61)	0.67 (0.18–2.56)	0.67 (0.18–2.56)
Q3 (n = 9, 115.6–135.9)	1.43 (0.47–4.37)	1.43 (0.43–4.72)	1.43 (0.43–4.72)
Q4 (n = 8, 136.0–220.9)	0.98 (0.29–3.29)	0.87 (0.33–3.48)	0.87 (0.22–3.48)

Model 1; adjusted for demographics (i.e. age, gender, race-ethnicity, education).

Model 2; adjusted for demographics & medical risk factors (i.e. age, sex, race-ethnicity, education, waist circumference, physical activity, moderate alcohol consumption, smoker, diabetes mellitus, systolic blood pressure, coronary artery disease, LDL, HDL).

Sensitivity analyses using the inverse probability weighted method to correct for potential selection bias that may have been introduced when the LpPLA2 cohort was selected from the full NOMAS cohort demonstrated qualitatively similar results (data not shown).

### Modification of the association of Lp-PLA2 by race-ethnicity

There was a non-significant trend toward effect modification of the association between LpPLA2 mass and all ischemic strokes by race-ethnicity (likelihood ratio test with 2 degrees of freedom, p = 0.14). The effect of LpPLA2 mass on the risk of IS was greater in non-Hispanic Whites (adjusted HR per SD 1.44, 95% CI 0.98–2.11), compared to Blacks (adjusted HR per SD 0.88, 95% CI 0.66–1.17) and Hispanics (adjusted HR per SD 1.01, 95% CI 0.79–1.28) (see table 4).

**Table 4:** Stratification by race ethnicity for all ischemic stoke.

Race-ethnicity group	HR* of LpPLA2-mass	95% CI of HR	
White	1.44	0.98	2.11
Black	0.88	0.66	1.17
Hispanic	1.01	0.80	1.28

adjusted for age, sex, race-ethnicity, education, waist circumference, physical activity, moderate alcohol consumption, smoking status, diabetes mellitus, systolic blood pressure, coronary artery disease, Low density lipoprotein, High density lipoprotein.

Each stroke etiology had different distributions of race-ethnicity. The proportion of strokes due to LAA was 24% among non-Hispanic Whites, 10% among non-Hispanic Blacks and 17% among Hispanics, whereas small vessel stroke was the etiologic subtype in 31% of non-Hispanic Blacks and 22% of Hispanics, but only 17% of non-Hispanic Whites. The proportion of cardioembolic stroke was 31% among non-Hispanic Whites, 46% among non-Hispanic Blacks and 33% among Hispanics.

The association of LpPLA2 mass with LAA stroke differed significantly by race-ethnicity (likelihood ratio test with 2 degrees of freedom, p = 0.01). LpPLA2 was associated with increased risk of LAA among non-Hispanic Whites (adjusted HR per SD 3.05, 95% CI 1.92–4.85), but not Blacks (adjusted HR per SD 1.19, 95% CI 0.70–2.05) or Hispanics (adjusted HR per SD 1.26, 95% CI 0.70–2.05) (see table 5). These interactions were not seen when analyzing LpPLA2 activity.

**Table 5:** Stratification by race ethnicity for large-artery atherosclerotic (LAA) stroke.

Race-ethnicity group	HR* of LpPLA2-mass	95% CI of HR	
White	3.05	1.92	4.85
Black	1.20	0.7	2.05
Hispanic	1.26	0.77	2.06

adjusted for age, sex, race-ethnicity, education, waist circumference, physical activity, moderate alcohol consumption, smoking status, diabetes mellitus, systolic blood pressure, coronary artery disease, Low density lipoprotein, High density lipoprotein.

## Discussion

In this urban multiethnic population-based sample we found that LpPLA2 mass levels previously associated with vascular disease risk are associated with risk of large artery atherosclerotic stroke, and the association was most prominent among non-Hispanic White participants, but not in other race-ethnic groups. We did not find an association of LpPLA2 with overall ischemic stroke risk.

There is limited data on the association of LpPLA2 levels and specific stroke etiologic subtypes. LpPLA2, a marker of inflammation and atherosclerosis, has been associated with coronary artery disease and myocardial disease. The causes of ischemic stroke are more heterogeneous than those of atherosclerotic heart disease, however, and considering all ischemic stroke as one disease for the purposes of these analyses may underestimate effects of particular risk factors. For example, several early epidemiologic studies indicated a lack of association between stroke risk and elevated lipid levels, but generally did not distinguish between stroke subtypes [16]. In recent studies, however, it has been demonstrated that lipids are risk factors specifically for large artery atherosclerotic strokes [17]. These findings supported our hypothesis that LpPLA2 may be associated with large artery atherosclerotic stroke risk, but not overall ischemic stroke risk. We did not find associations of LpPLA2 mass levels with lacunar strokes, arising from lipohyalinosis of small perforating arteries, which are generally considered to be related to hypertension and diabetes, or with cardioembolic stroke, related to the formation of thrombin-rich clots that form in the heart, often in the presence of atrial fibrillation. Further indirect evidence in support of this hypothesis may be drawn from a recently published study of a population of patients with transient ischemic attacks in which LpPLA2 activity was increased specifically when LAA was the most likely mechanism for the TIA [18]. Moreover, others have shown that LpPLA2 expression was significantly higher in the carotid plaque of symptomatic patients, in particular in those with TIA [19].

We have previously reported that LpPLA2 mass as well as activity levels in the top quartile predict recurrent stroke after first ischemic stroke [8]. These findings seem not to be consistent with the lack of association of LpPLA2 with ischemic stroke risk in the present study. However, the effect of LpPLA2 on the risk of recurrent stroke appeared to be greater for those with previous atherosclerotic strokes [8]. Moreover, the population of incident stroke patients differs from the stroke-free population, such that subjects with a first stroke are likely in a very high vascular risk category compared to the stroke free population. Therefore the predictive value of LpPLA2 may differ.

We also found that the association of LpPLA2 with risk of atherosclerotic stroke differed by race-ethnic group; the effect of LpPLA2 on LAA risk was greater among non-Hispanic Whites compared to non-Hispanic Blacks or Hispanics. It thus may be that risk factors for large vessel atherosclerosis are not exactly the same in all ethnicities, or that the effect of other risk factors, like diabetes or hypertension, are more important than LpPLA2 in other race-ethnic groups. Studies on the value of LpPLA2 mass and/or activity levels in the prediction of stroke risk have been carried out predominantly in Caucasian populations, and differences in LpPLA2 across race-ethnicities have been reported [20]. Of the four largest population studies, including the Rotterdam study cohort [21], the Malmö Diet and Cancer Study (MDCS) [22], the Women's Health Initiative Observational Study (WHI-OS) [23] and the Atherosclerosis Risk in Communities (ARIC) [24] study, only ARIC and WHI-OS included a relatively large proportion of Blacks. Hispanics have not been studied with regard to LpPLA2 and stroke risk. Although similar well-known vascular risk factors and molecular pathways are involved in the global process of atherogenesis, local expression and severity of atherosclerosis differs among vulnerable individuals. A paradigm of this disparity is the difference in race-ethnic distribution of atherosclerosis. Carotid bifurcation atherosclerosis, for example, appears to be most prevalent among Caucasians compared to African Americans, Hispanics or Asians [25]–[29]. Our study is the first assessing LpPLA2 as a potential risk factor for incident ischemic stroke events in a multiethnic cohort including a large proportion of Hispanics. This large proportion of Hispanics may explain why LpPLA2 levels were not associated with all ischemic stroke in our cohort and why in contrast in other primarily European Caucasian populations in which LAA may be a relatively more prominent subtype [30] investigators found an association of LpPLA2 with all ischemic stroke.

Stroke etiology in the present cohort differed by race-ethnicity. Whites were more likely to have LAA than Blacks or Hispanics, but less likely to have small vessel disease than other race-ethnicity. These distributions were consistent with other studies. In the South London Ethnicity and Stroke study, the proportion of LAA was greater among Whites (14.3%) than among Blacks (8%) whereas small vessel disease was much more common among Blacks (33%) compared to Whites (14%) [25]. In the Rochester Epidemiology Project the incidence of stroke due to large-vessel atherosclerosis with stenosis was significantly greater among Whites (27 per 100 000) than Blacks (17 per 100 000) [31].

We found associations for LpPLA2 mass levels but not activity levels. We previously found that mass levels were more strongly associated than activity levels with outcomes after incident stroke in our cohort [8], [9]. LpPLA2 activity is also more strongly associated with lipid markers then mass [4] and, as we previously reported, baseline lipid panel components were not clearly associated with an increased stroke risk in this cohort [32], possibly because of temporal trends in use of statins and levels of lipids. Further possible reasons for differences between mass and activity levels as prognostic markers include acute changes in activity levels in the setting of lipoprotein changes.

Our study has limitations. First, the blood samples were stored at −80 C for several years, which could potentially lead to some protein degradation. However, LpPLA2 seems to be quite stable over time when stored at −70 C [33]. Moreover our samples were stored for a similar length of time as compared to other studies [4]. Second, we did not have blood available for analysis in all participants in our cohort, but the differences between our entire cohort and the sample analyzed in this study were minimal. Third, repeated measurements of LpPLA2 and measurements closer to the event or their change over time may be a better or of ischemic stroke than levels measured at a single baseline point. Fourth, there are shortcomings with all etiological classifications of ischemic stroke and it is always difficult to determine stroke subtype with certainty. In the NOMAS cohort we tried to minimize misclassification by relying only on a consensus among vascular neurologists, with a third neurologist adjudicating if needed [14]. If misclassification occurred it would rather be non-differential misclassification due to missed cases thus underestimating the association of LpPLA2 and LAA strokes. Fifth, self-report of race-ethnicity depends on cultural and social characteristics and is not equivalent to a biological marker or genetic indicator. Finally, as the number of LAA strokes is relatively small, analyses by race-ethnicity groups should be considered with caution and differences observed may be due to chance. Although the magnitude of association may be over- or underestimated, and thus the precision of the point estimates may be limited, we are confident that the associations we found are reliable. But these results need external validation.

The strengths of this study include the population-based multiethnic cohort, including a large proportion of Hispanics who are frequently underrepresented in other cohort studies, minimal loss to follow-up, and the ability to adjust for numerous potential covariates. Moreover, we were able to assess stroke etiology and potential interactions, and we ran sensitivity analyses to exclude biases due to selection of the subsample.

If our results are confirmed, this could have potential clinical implications. For example it may be that stroke risk reduction strategies aimed at targeting LpPLA2 inhibition will have differential benefits across different ethnicities. Concerning potential primary prevention trials with the LpPLA2 inhibitor darapladib it will be important to adequately select patients who are most likely to benefit.

In conclusion, our study aimed at investigating the association of LpPLA2 with incident ischemic stroke in a multiethnic cohort. LpPLA2 mass levels were associated with risk of atherosclerotic stroke among non-Hispanic White participants, but not in other race-ethnic groups in this cohort. Further study is needed to confirm these race-ethnic differences and the reasons for them.
